# Lead-Free Halide Perovskite Cs_2_AgBiBr_6_/Bismuthene Composites for Improved CH_4_ Production in Photocatalytic CO_2_ Reduction

**DOI:** 10.1021/acsaem.2c03105

**Published:** 2023-02-01

**Authors:** Michael Segundo Sena, Junyi Cui, Yasmine Baghdadi, Eduardo Rattner, Matyas Daboczi, André Luís Lopes-Moriyama, Andarair Gomes dos Santos, Salvador Eslava

**Affiliations:** † Department of Graduation in Chemical Engineering, Universidade Federal do Rio Grande do Norte/UFRN, 59.078-970Rio Grande do Norte, Brazil; ‡ Department of Chemical Engineering, Imperial College London, SW7 2BX, London, United Kingdom; § Department of Agrotechnology and Social Sciences, Universidade Federal Rural do Semi-Árido/UFERSA, 59.600-000Rio Grande do Norte, Brazil

**Keywords:** halide perovskites, 2D bismuthene, solar fuels, photocatalytic CO_2_ reduction, CH_4_ production, charge separation

## Abstract

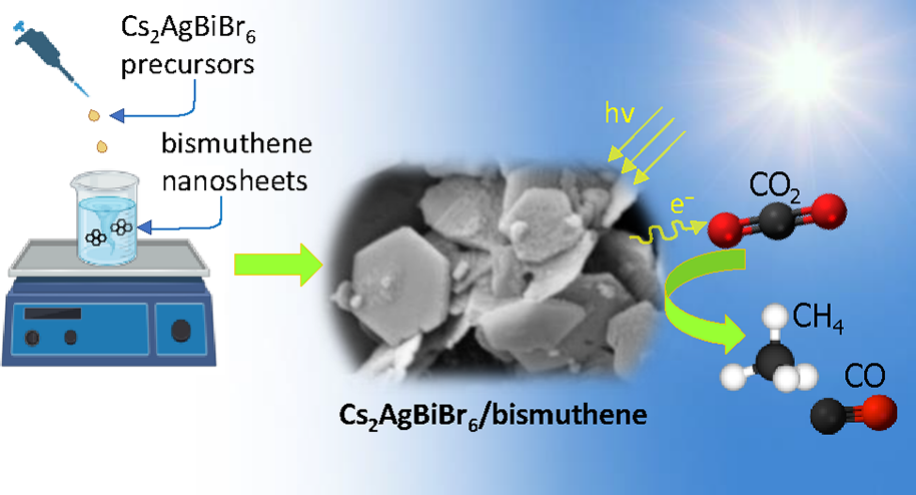

CO_2_ photocatalytic conversion into value-added fuels through solar energy is a promising way of storing renewable energy while simultaneously reducing the concentration of CO_2_ in the atmosphere. Lead-based halide perovskites have recently shown great potential in various applications such as solar cells, optoelectronics, and photocatalysis. Even though they show high performance, the high toxicity of Pb^2+^ along with poor stability under ambient conditions restrains the application of these materials in photocatalysis. In this respect, we developed an in situ assembly strategy to fabricate the lead-free double perovskite Cs_2_AgBiBr_6_ on a 2D bismuthene nanosheet prepared by a ligand-assisted reprecipitation method for a liquid-phase CO_2_ photocatalytic reduction reaction. The composite improved the production and selectivity of the eight-electron CH_4_ pathway compared with the two-electron CO pathway, storing more of the light energy harvested by the photocatalyst. The Cs_2_AgBiBr_6_/bismuthene composite shows a photocatalytic activity of 1.49(±0.16) μmol g^–1^ h^–1^ CH_4_, 0.67(±0.14) μmol g^–1^ h^–1^ CO, and 0.75(±0.20) μmol g^–1^ h^–1^ H_2_, with a CH_4_ selectivity of 81(±1)% on an electron basis with 1 sun. The improved performance is attributed to the enhanced charge separation and suppressed electron–hole recombination due to good interfacial contact between the perovskite and bismuthene promoted by the synthesis method.

## Introduction

The need to tackle global warming, largely resulting from copious amounts of greenhouse gases released into the atmosphere from the burning of fossil fuels, has never been more pressing. According to the European Union Commission for climate action, CO_2_ produced by human activity is the largest contributor to global warming.^1^ The global average temperature has already risen by 1.1 °C above preindustrial (pre-1750) levels and may rise up to 2.1–3.5 °C by 2100 unless tougher mitigation action is taken.^2^ Moreover, the natural cycle of carbon is being disrupted by human activities such as deforestation and degradation of tropical forests, which already account for roughly 12% of total anthropogenic greenhouse gas emissions and dominate the national CO_2_ emission profile of developing countries such as Brazil and Indonesia.^3^

The endergonic conversion of CO_2_ into value-added chemical fuels (e.g., CO, CH_4_, CH_3_OH) is one of the most promising ideas to address global warming and store renewable energy.^4^ While the task of converting CO_2_ into fuel is scientifically challenging, it would have significant benefits, as it provides a way to use an abundant and renewable substance in nature to produce fuels and feedstocks for which the current industry infrastructure is already adapted for use. Recently, many techniques such as photoelectrochemical (PEC),^5^ thermocatalytic,^6^ biochemical,^7^ electrocatalytic,^8^ and photocatalytic^9^ techniques have made considerable advances in this area. Among them, photocatalytic CO_2_ conversion is an attractive and promising technology because it offers a potentially affordable, carbon-free route to the synthesis of solar fuels.^10^

Generally, the routes for solar-driven CO_2_ reduction include two-electron-reduction (*E*° = −0.53 V_SHE_ for CO, *E*° = −0.61 V_SHE_ for HCOOH), four-electron-reduction (*E*° = −0.48 V_SHE_ for HCHO), six-electron-reduction (*E*° = −0.38 V_SHE_ for CH_3_OH) and eight-electron-reduction reactions (*E*° = −0.24 V_SHE_ for CH_4_).^11^ Among those reaction processes, CH_4_, with an enthalpy of combustion of −890.03 kJ mol^–1^, can store the most solar energy into chemical energy for further utilization. However, most of the existing photocatalysts have insufficient reduction ability for the eight-electron CH_4_ production pathway.^12,13^

Semiconductor photocatalysts have been extensively reported in recent years in many applications such as organic compound degradation,^14^ H_2_ evolution,^15^ air purification,^16^ and CO_2_ reduction reactions.^17^ The photocatalytic CO_2_ reduction is often referred to as “artificial photosynthesis” due to its potential for reducing CO_2_ on a semiconductor surface by utilizing the energy of the sun and protons from water to produce hydrocarbons, thus mimicking the natural process carried out by plants. It has great potential to produce value-added fuels and provide an interesting route to tackle both climate change and renewable energy production.^18^

The CO_2_ conversion to CH_4_ is not a straightforward process, as there are thermodynamic and kinetic limitations to the reaction. An efficient semiconductor photocatalyst for this kind of reaction should have a conduction band edge with a lower potential than that of the target reduction reaction, the ability to absorb a wide energy range of solar irradiation, high mobility, and efficient separation of the photogenerated charges.^19^

Among many types of semiconductor materials for photocatalytic applications, halide perovskites have recently emerged as promising photocatalysts for CO_2_ reduction due to their excellent properties including tunable band structure, high absorption coefficient, and excellent charge separation efficiency.^20^ In recent years, the all-inorganic perovskite CsPbBr_3_ has attracted great attention for photocatalytic CO_2_ reduction. Hou et al. prepared CsPbBr_3_ quantum dots for photocatalytic reduction of CO_2_ to CO and CH_4_, with productions of 4.3 and 1.5 μmol g^–1^ h^–1^, respectively.^21^

Despite the promising photocatalytic properties of CsPbBr_3_, the use of a material composed of toxic Pb^2+^ cations is not sustainable, hindering its development and application.^22^ Alternatively, lead-free halide perovskites have recently been studied to overcome the toxicity problem. Examples of lead-free perovskites are CsSnX_3_, CsSbX_3_, and various double perovskites such as Cs_2_AgBiBr_6_ (DP). They show a band gap of 1.8–2.2 eV and a long carrier recombination lifetime. Among various Pb-free double perovskites, Cs_2_AgBiBr_6_ has shown better stability toward moisture, air, heat, and light.^23^

Bismuth has recently been reported as a highly efficient electrocatalyst for the CO_2_ reduction reaction. As a stable freestanding two-dimensional (2D) bismuth monolayer, bismuthene (Bi) has demonstrated high electrocatalytic efficiency for formate (HCOO^–^) production from the CO_2_ reduction reaction.^24^ In a recent work, 2D bismuthene has been used as a functional interlayer between BiVO_4_ and NiFeOOH for enhanced oxygen-evolution photoanodes, significantly improving the PEC performance of photoanodes by optimizing the separation and mobility of photoinduced charges.^25^

In this work, we have adopted a ligand-assisted reprecipitation (LARP) method to successfully synthesize, for the first time, Cs_2_AgBiBr_6_/bismuthene (DP/Bi) composites for photocatalytic CO_2_ reduction to CH_4_. Our approach promotes the self-assembly of the perovskite nanoparticles on the bismuthene nanosheets, which influences perovskite nucleation and growth, forming hierarchical hexagonal plates. The DP/Bi heterostructure photocatalysts showed significant improvement when compared to a pristine double perovskite. The photocatalytic CO_2_ reduction achieved 1.49(±0.16) μmol g^–1^ h^–1^ CH_4_, 0.67(±0.14) μmol g^–1^ h^–1^ CO, and 0.75(±0.20) μmol g^–1^ h^–1^ H_2_, with a CH_4_ selectivity of 81(±1)% on an electron basis with 1 sun of simulated light. The improved performance was attributed to enhanced charge separation and suppressed electron–hole recombination achieved through a good interfacial contact between the perovskite and bismuthene promoted by the synthesis method.

## Experimental Section

### Chemicals

CsBr (99.9%, Acros Organics), AgBr (99%, Sigma-Aldrich), BiBr_3_ (99%, Sigma-Aldrich), BiCl_3_ (99.9%, Sigma-Aldrich), NaBH_4_ (99%, Sigma-Aldrich), ethyl acetate (99.5%, Sigma-Aldrich), oleic acid (99%, Sigma-Aldrich), 2-ethoxyethanol (99.8%, Sigma-Aldrich), *N*,*N*-dimethylformamide (99,8%, Sigma-Aldrich), ethanol (99.5%, Sigma-Aldrich), and methanol (99.8%, Sigma-Aldrich) used in this work are of analytical grade and were used without further purification.

### Synthesis of Cs_2_AgBiBr_6_ (DP) Nanoparticles

**Figure 1:**
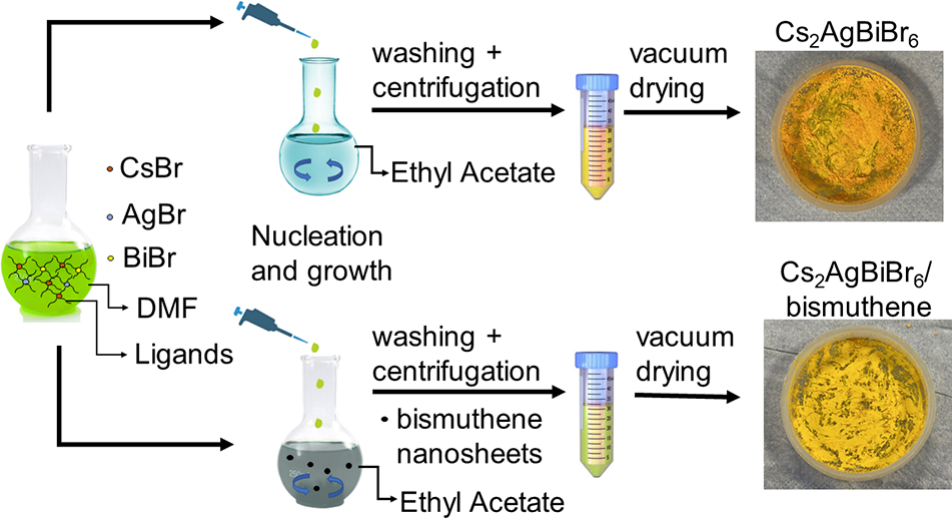
Schematic illustration of the synthetic procedure of Cs_2_AgBiBr_6_ nanoparticles and Cs_2_AgBiBr_6_/bismuthene composites.

An optimized LARP protocol based on the work by Ng et al.^26^ was adopted to synthesize Cs_2_AgBiBr_6_ nanoparticles as a reference. The LARP reaction is depicted in Figure 1. Briefly, 25 mL of an anhydrous dimethylformamide (DMF) precursor solution was prepared to contain 1 mmol of CsBr, 0.5 mmol of AgBr, 0.5 mmol of BiBr, and 10 vol % of oleic acid and heated to 80 °C with continuous stirring to ensure a homogeneous solution. The resulting solution was left to cool to room temperature. A 25 mL portion of the obtained mixture was swiftly injected into 250 mL of ethyl acetate under vigorous stirring. After stirring, the resulting suspension was centrifuged for 10 min at 4500 rpm. After centrifugation, the supernatant was discarded, and the precipitate was washed twice with ethyl acetate. The powders were then vacuum-dried at 60 °C for 12 h.

### Synthesis of Bismuthene (Bi)

Bismuthene nanosheets were synthesized by a wet chemical method as reported by Yang et al.^24^ BiCl_3_ (1.5 mmol) was dissolved in 50 mL of 2-ethoxyethanol, followed by ultrasonication of the mixture to form a uniform and transparent solution. The solution was vigorously stirred for 30 min in an oil bath at 120 °C while N_2_ was bubbled into the solution to maintain an inert atmosphere. The solution was left to cool to room temperature. A 20 mL portion of NaBH_4_ (60 mmol) was then added dropwise to the bismuth solution, still under a nitrogen atmosphere, with stirring for 5 min under these conditions. The resultant black precipitate was collected by centrifugation and washed twice with DI water and ethanol, respectively. The obtained powder was dried in a vacuum oven at room temperature for 24 h and then stored under an N_2_ atmosphere for later characterization or application.

### Synthesis of Cs_2_AgBiBr_6_/Bismuthene (DP/Bi) Composites

We adapted the LARP protocol to self-assemble Cs_2_AgBiBr_6_ nanoparticles onto bismuthene nanosheets as illustrated in Figure 1. Different amounts of bismuthene (2.5, 5, and 10 mg) were added to 250 mL of ethyl acetate, and the suspension was then sonicated for 24 h to exfoliate it in thin layers. A 25 mL portion of the DP precursor solution in DMF was prepared as mentioned in the previous section, which was then swiftly injected into the bismuthene suspension in ethyl acetate with vigorous magnetic stirring for 30 min. The precipitates were collected by centrifugation at 4500 rpm for 10 min. The powders were washed twice with ethyl acetate and dried in a vacuum oven at 60 °C for 12 h. The composites with nominal weight percentages of bismuthene of 0.5, 1, and 2% in the composition were referred to as DP/Bi0.5, DP/Bi1, and DP/Bi2, respectively.

### Characterization

Powder X-ray diffraction (XRD) was carried out with a X’Pert PRO diffractometer by PANalytical operated at 40 kV voltage and 40 mA current using Cu Kα (λ = 0.15418 nm) radiation in the 2θ range 10–50°. The coherent diffraction domain sizes of Cs_2_AgBiBr_6_ and its composites were calculated using the Scherrer equation on the peaks corresponding to the (022), (040), and (044) planes. X-ray photoelectron spectroscopy (XPS) analysis was done using a Thermo Fisher K-Alpha spectrometer and monochromated Al Kα_1_ X-ray source (1593 eV), and the pass energy was set at 20 eV with constant pass energy (CPE) mode. Furthermore, the binding energies were referenced relative to the C (1s) C***–***C bond at 284.8 eV. The XPS data were processed using the software Thermo Avantage version 5.9922. The morphology and crystallinity of the as-prepared samples were examined by high-resolution transmission electron microscopy (HRTEM) using a JEOL JEM-2100Plus microscope operating at an accelerating voltage of 200 kV. The morphological aspects were also examined by scanning electron microscopy (SEM) on a Zeiss Auriga Cross Beam microscope. The light absorption of the as-prepared samples was measured by UV–visible diffuse reflectance spectroscopy (UV–vis DRS) on a Shimadzu 2600 spectrophotometer using an integrating sphere for diffuse reflectance and BaSO_4_ as a standard. The baseline was set using pure BaSO_4_, and then 10 mg of a sample was mixed with BaSO_4_ for analysis. Photocurrent measurements were carried out in a three-electrode PEC cell with a quartz window, a working electrode, a Pt-wire counter electrode, a nonaqueous Ag/AgCl reference electrode solution, and 0.1 M tetrabutylammonium hexafluorophosphate (TBAPF6) in acetonitrile as an electrolyte solution.^27^ A 300 W Xe lamp equipped with an AM1.5G filter (LOT Quantum Design) was used at 100 mW cm^–2^ (1 sun) which was chopped (10 s off, 10 s on, repeated three times) with an automatic shutter. An external potential of +0.6 or −0.6 V vs the Ag/AgCl reference electrode was applied with a potentiostat (Ivium CompactStat).

### Photocatalytic CO_2_ Reduction Test

The photocatalytic CO_2_ reduction tests were carried out under simulated solar irradiation at room temperature in a gastight 85 mL stainless steel reactor with a PTFE lining and a top quartz window. A 300 W Xe lamp equipped with an AM1.5G filter was placed vertically above the reactor, and the light intensity was adjusted to 1 sun (100 mW cm^–2^). A 7 mg portion of a powder sample was dispersed in 35 mL of anhydrous methanol and sonicated for 2 min to create a well-dispersed suspension before loading into the photoreactor. Before irradiation, the photoreactor was purged with He gas for 1 h to remove air from the system. Then, CO_2_ gas was bubbled through the suspension for 1 h at 30 mL min^–1^ with continuous stirring to saturate the system. The gas inlet and outlet in the reactor were closed before starting the irradiation for 2 h under 1 simulated sun. The products were analyzed by gas chromatography (GCMS, Shimadzu GC-2030 Plus) with He as a carrier gas and a barrier ionization discharge (BID) detector. The selectivity (*S*) of the products was calculated on a total electron consumption basis, as given in eqs 1–3^22^
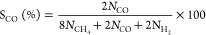
1
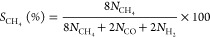
2
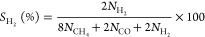
3where *N*_CH_4__*, N*_CO_, and *N*_H_2__ are the production rates of CH_4_, CO, and H_2_ in μmol g^–1^ h^–1^ and the coefficients 8, 2, and 2 are used to account for the total electrons involved in the CO_2_ reduction to form CH_4_, CO, and H_2_, respectively. The apparent quantum efficiency (AQE) was measured using the same experimental setup, but with a 450 nm LED light source to obtain monochromatic light. The AQE was calculated according to eq 4:
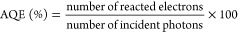
4

A series of control experiments were carried out to confirm the CO_2_ reduction reaction, consisting of experiments (1) in darkness, (2) without photocatalyst, and (3) in He. In experiment 1 the same photocatalytic procedure was performed using DP/Bi0.5 as a photocatalyst without light irradiation. In experiment 2, anhydrous methanol was loaded into the photoreactor without any photocatalyst. In experiment 3 only He gas was used to purge the photoreactor containing the methanol suspension with DP/Bi0.5 as a photocatalyst.

## Results and Discussion

**Figure 2:**
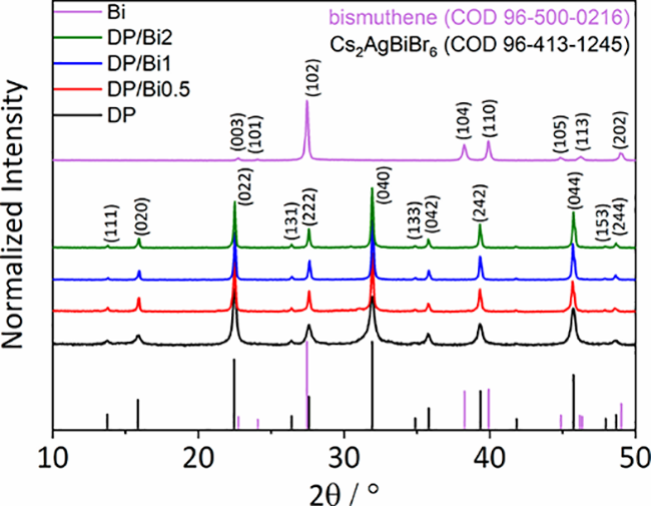
XRD patterns of bismuthene (Bi), Cs_2_AgBiBr_6_ (DP), and DP/Bi composites.

The XRD patterns of the as-prepared DP nanoparticles, few-layered bismuthene, and composites with varying contents of bismuthene (from 0.5 to 2 wt %) are shown in Figure 2. First, the XRD patterns of DP were assigned to the cubic phase of Cs_2_AgBiBr_6_ consistent with the standard pattern of the Crystallography Open Database (COD) Card No. 96-413-1245, with *a* = 11.2818 Å and space group symmetry *Fm*3̅*m*. The obtained XRD patterns of DP show its characteristic diffraction at 13.7, 15.9, 22.5, 26.4, 27.6, 31.9, 34.6, 35.7, 39.3, 45.7, 47.9, and 48.6° (2θ), corresponding to the (111), (020), (022), (131), (222), (040), (133), (042), (242), (044), (153), and (244) planes, respectively. Second, the XRD patterns of the few-layered bismuthene prepared by a wet chemical method were indexed with a hexagonal crystal structure (COD Card No. 96-500-0216), with *a* = *b* = 4.5488 Å, *c* = 11.8683 Å, and space group *R*3*®m*. The obtained XRD pattern of bismuthene shows its characteristic diffraction peaks at 22.7, 24.1, 27.4, 38.2, 39.9, 44.8, 46.2, and 48.9° (2θ), corresponding to the (003), (101), (102), (104), (110), (105), (113), and (202) planes, respectively. Finally, the XRD patterns of DP/Bi composites all show the same cubic Cs_2_AgBiBr_6_ crystal phase, which indicates that the type of crystal phase is not affected by the addition of bismuthene under this synthesis condition. No bismuthene peaks could be identified on the XRD patterns of the composites, confirming its good dispersion in the composite and its low weight percentage in the composition. The XRD analysis confirmed that the LARP method used to synthesize the DP and DP/Bi samples was a successful approach, which we also found relatively easy to carry out and reproduce.

The coherent diffraction domain size of the DP and DP/Bi composites were calculated using the Scherrer equation.^28,29^ The domain size of the bare perovskite (DP) was 21.2(±4.1) nm. Therefore, the LARP method and oleic acid ligands prevented large domain size formation but kept sizes above 10 nm, avoiding quantum size effects that increase the band gap.^30^ The coherent diffraction domain size of the perovskite increased with the increasing bismuthene content, from 21.2(±4.1) nm for bare DP to 40.2(±4.0), 45.8(±5.7), and 48.7(±4.4) nm for DP/Bi0.5, DP/Bi1, and DP/Bi2, respectively. This increase in domain size is evidence that 2D bismuthene influenced the nucleation and growth of the perovskite crystals.

**Figure 3:**
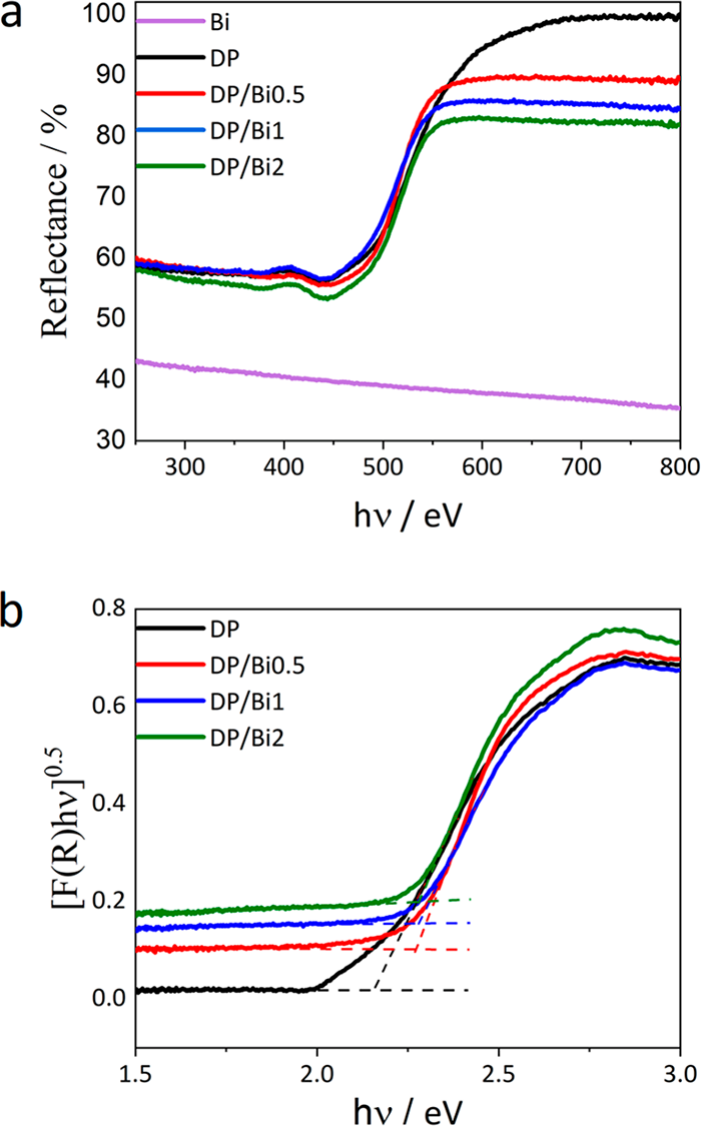
(a) Diffuse reflectance spectra of Bi, DP, DP/Bi0.5, DP/Bi1, and DP/Bi2. (b) Tauc plots of [*F*(*R*)*h*ν]^0.5^ as a function of the photon energy (*h*ν) for DP, DP/Bi0.5, DP/Bi, and DP/Bi2.

The UV–vis light absorption of the as-prepared samples was tested, and the results are presented in Figure 3. The diffuse reflectance (DR) spectrum of bismuthene indicates a wide absorption in the broad UV–vis range, in agreement with its metallic character (Figure 3a). The DR spectra of the composite samples show an increase of absorption in a wide range of energies with an increase of bismuthene content; the DR spectra of the perovskite samples (bare and composites) have similar shapes but are shifted to lower reflectance values throughout the entire spectral range. An absorption edge is present in all the DP-containing samples at around 550 nm, assigned to the band gap of the DP Cs_2_AgBiBr_6_, which is in agreement with the literature on the Cs_2_AgBiBr_6_ double perovskite.^31,32^ The Tauc equation^33^ was used to represent the solar absorption and evaluate the band gap (*E*_*g*_) of Cs_2_AgBiBr_6_ in bare and composite samples. The plots of [*F*(*R*)*h*ν]^0.5^ as a function of the photon energy (*h*ν) are shown in Figure 3b, where *F*(*R*), *h*, and ν are the Kubelka–Munk function, Planck’s constant, and the light frequency, respectively. The exponent 0.5 represents the indirect electronic transition of the semiconductor Cs_2_AgBiBr_6_. The band gap of the samples was estimated by measuring the interception of the extrapolation of the linear part of the curve and its baseline; accordingly, the estimated band gaps are 2.14, 2.27, 2.28, and 2.28 eV for DP, DP/Bi0.5, DP/Bi1, and DP/Bi2, respectively. Similar band gap values are obtained with a variation within the error of such measurement where scattering effects can have an influence. The same band gap values agree with the absence of quantum size effects and the detection of the same crystalline phase by XRD (Figure 2).

**Figure 4:**
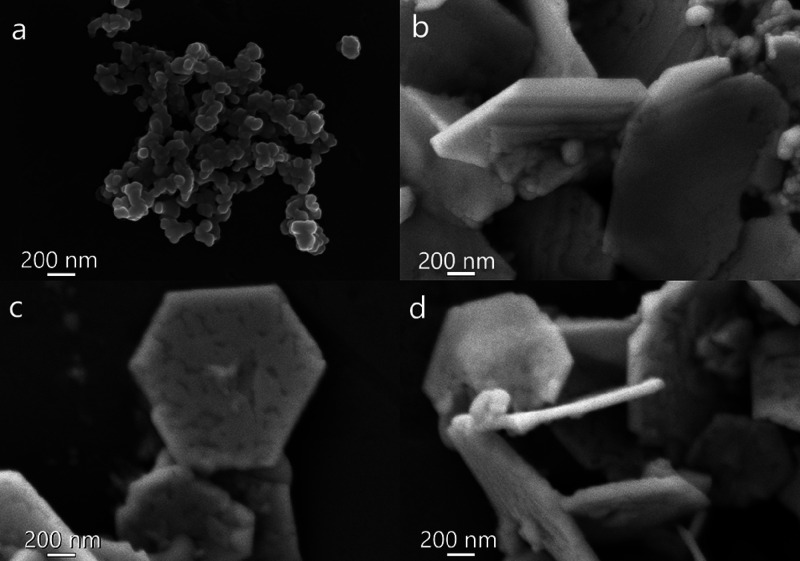
SEM micrographs of (a) DP, (b) DP/Bi0.5, (c) DP/Bi1, and (d) DP/Bi2.

**Figure 5:**
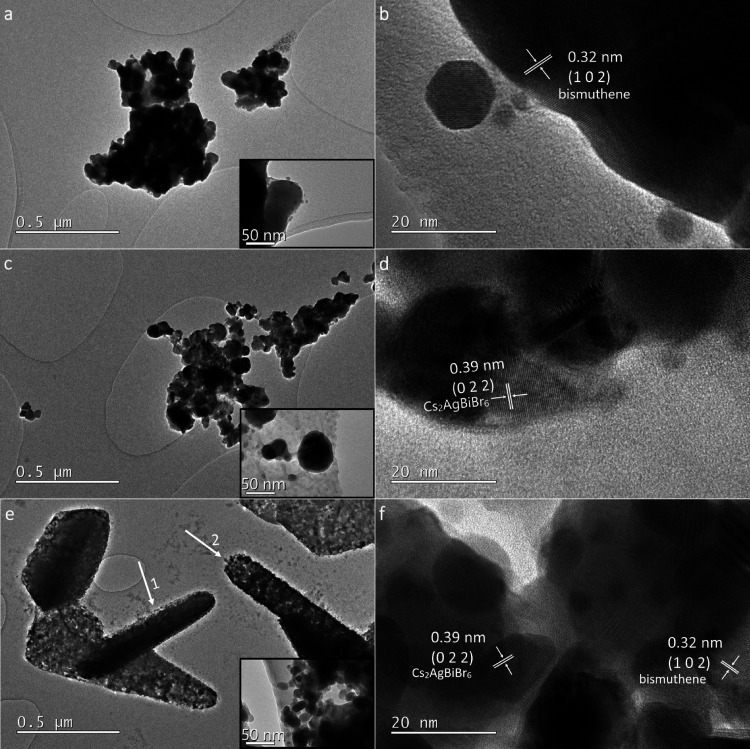
TEM (left) and HRTEM (right) micrographs of (a, b) bismuthene, (c, d) DP, and (e, f) DP/Bi1.

To characterize the morphology of the samples, an SEM analysis was conducted. Figure 4a–d shows the micrographs of the DP, DP/Bi0.5, DP/Bi1 and DP/Bi2 samples, respectively. The SEM micrograph of the DP sample (Figure 4a) exhibits round-shaped nanoparticles in isolated and agglomerated forms, with diameters ranging from 65 to 87 nm. The micrographs of the composites display a very different morphology compared to the nanoparticles of the pristine perovskite. Figure 4b–d shows large plates of hexagonal morphology ranging in length from 1.3 to 2.1 μm and thickness ranging from 48 to 94 nm. These hexagonal microparticles are formed by the self-assembly of the Cs_2_AgBiBr_6_ nanoparticles on bismuthene nanosheets, as further discussed in the TEM characterization. The morphology and crystal structure of the samples were also examined by TEM and high-resolution TEM (HRTEM). The TEM micrograph of bismuthene (Figure 5a) shows irregular sheet-like morphologies of various sizes. The almost transparent regions on the micrographs suggest that ultrathin nanostructures were achieved. Darker color regions can be ascribed to the crumpling and stacking of bismuthene nanosheets.^34^ The HRTEM image of bismuthene is displayed in Figure 5b, where clear lattice fringes can be observed, and the spacing of the measured fringes is 0.32 nm, assigned to the (102) plane of the hexagonal crystal phase of bismuthene. Figure 5c shows a TEM micrograph of the pristine perovskite DP nanoparticles with sizes that vary from 10 to 80 nm. The HRTEM lattice fringes show an interplanar spacing of 0.39 nm, which is assigned to the (022) plane of the Cs_2_AgBiBr_6_ cubic phase (Figure 5d). Figure 5e shows the self-assembled hierarchical structures of the DP/Bi1 composite, where the hexagonal plate structures can be observed from different orientations. The arrows 1 and 2 in Figure 5e point to the side view of the hexagonal plates measuring approximately 152 and 151 nm, respectively, in thickness. The higher magnification in the inset of Figure 5e clearly shows that the hexagonal hierarchical structures are formed by perovskite nanoparticles assembled together. The HRTEM micrograph of DP/Bi1 (Figure 5f) shows lattice fringes in the nanoparticles with an interplanar spacing of 0.39 nm attributed to the (022) crystal plane of the Cs_2_AgBiBr_6_ phase. There is also the presence of lattice fringes in the nanosheet structure over the nanoparticles measuring an interplanar spacing of 0.32 nm corresponding to the (102) plane of the bismuthene phase. This is evidence of intimate contact between the perovskite and bismuthene surfaces.

These bismuthene nanosheets obviously have a major role in the directionality of the self-assembly process of the perovskite nanoparticles into the hexagonal hierarchical structure. However, the exact mechanism of the directional self-assembly is not yet fully understood. It is well established in the literature that the use of ligands such as oleic acid in the synthesis of nanoparticles enables better control of the nanocrystal size and crystallization.^35,36^ As a surfactant it provides the steric stabilization of the nanoparticles against van der Waals attractive interactions and thereby prevents their agglomeration. It also limits the growth of nanoparticles and prevents an Ostwald ripening process from taking place, since its surface layers act as a barrier to mass transfer.^37^ These characteristics may facilitate the process of self-assembly of the nanoparticles in hierarchical structures.

For the synthesis of the bare perovskite, the precursors are dissolved in a *good* solvent (DMF) containing the oleic acid ligand and undergo a swift change of solubility by several orders of magnitude when injected into a *poor* solvent (ethyl acetate), which results in the rapid nucleation and formation of the Cs_2_AgBiBr_6_ nanoparticles. For the composite synthesis, the bismuthene nanosheets are already well dispersed and exfoliated in the ethyl acetate solvent after 24 h of ultrasonication. When the perovskite precursor solution is injected into the bismuthene-ethyl acetate dispersion, bismuthene nanosheets can act as nucleation sites, influencing the crystal growth and resulting in the hexagonal hierarchical plate structures.

**Figure 6:**
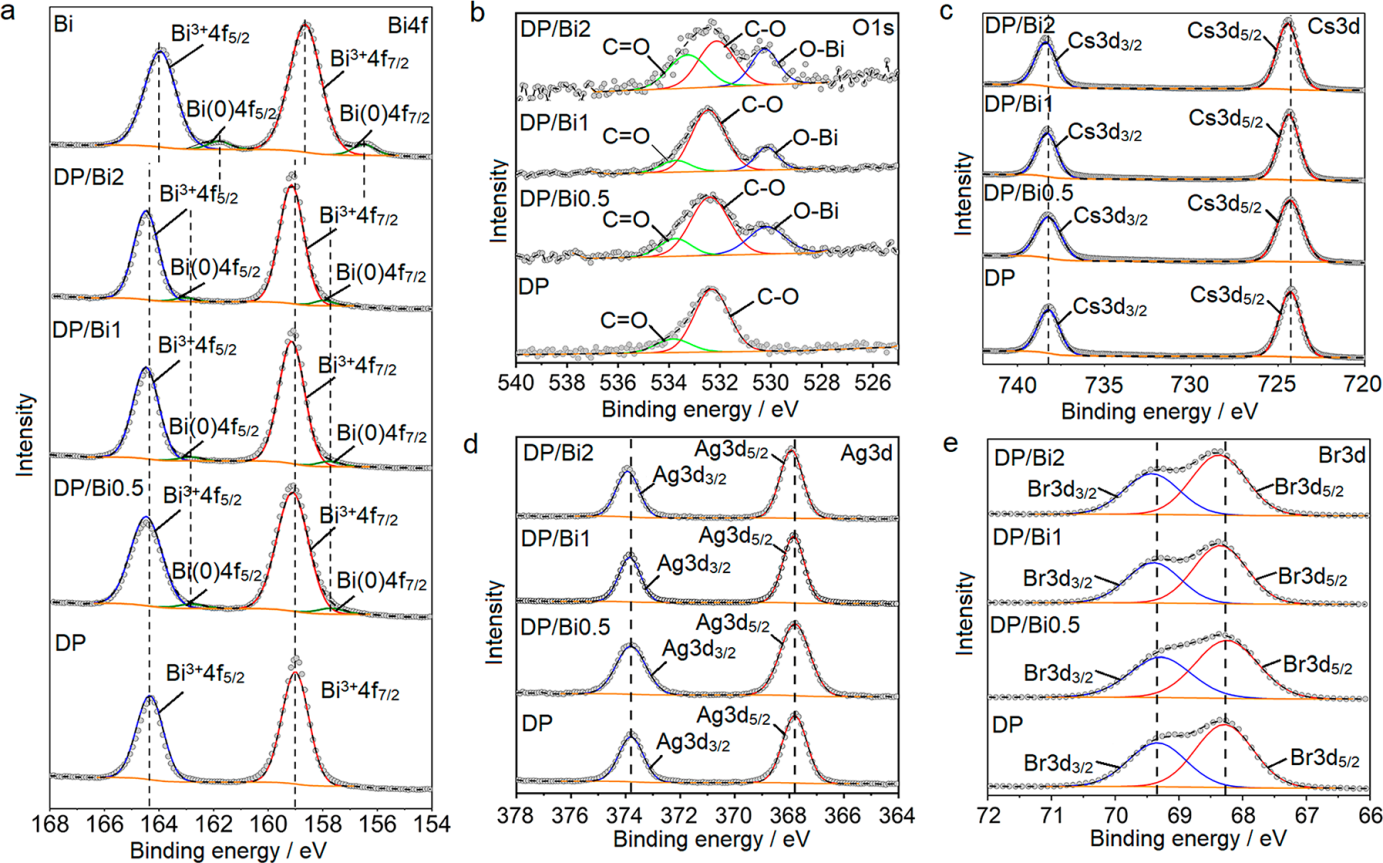
XPS spectra of DP, DP/Bi0.5, DP/Bi1, DP/Bi2, and Bi: (a) Bi 4f; (b) O 1s; (c) Cs 3d; (d) Ag 3d; (e) Br 3d.

In order to examine the chemical environment and the oxidation state of the atoms on the surface of the samples, X-ray photoelectron spectroscopy (XPS) characterizations were conducted. The survey spectra of the DP (Figure S1) and of a representative DP/Bi1 sample (Figure S2) indicate the presence of Cs, Ag, Bi, Br, C, and O. Figure 6a–e shows the high-resolution Cs 3d, Ag 3d, Bi 4f, Br 3d, and O 1s spectra of Bi, DP, and the DP/Bi composites. The peak positions of the high-resolution spectra of the samples can be seen in Table 1.

**Table 1:** Peak Positions (in eV Binding Energy) in the XPS Spectra of the Bi, DP, DP/Bi0.5, DP/Bi1, and DP/Bi2 Samples

	peak position				
transition	Bi	DP	DP/Bi0.5	DP/Bi1	DP/Bi2
Cs 3d_5/2_		724.29	724.31	724.37	724.45
Cs 3d_3/2_		738.20	738.20	738.29	738.37
Ag 3d_5/2_		367.80	367.79	367.85	367.93
Ag 3d_3/2_		373.80	373.80	373.85	373.92
Bi^3+^ 4f_7/2_	158.64	159.00	159.11	159.13	159.14
*Bi^3+^ 4f_5/2_*	163.95	164.33	164.46	164.48	164.47
Bi^0^ 4f_7/2_	156.49		157.75	157.76	157.95
Bi^0^ 4f_5/2_	161.80		162.86	162.87	163.06
Br 3d_5/2_		68.28	68.25	68.35	68.37
Br 3d_3/2_		69.34	69.29	69.40	69.42
C 1s C–C	284.80	284.80	284.80	284.80	284.80
C 1s C–O	286.33	286.38	286.51	286.37	286.34
C1s C=O	288.37	289.38	288.88	289.00	287.30

The high-resolution Bi 4f spectra of Bi, DP and DP/Bi composites are shown in Figure 6a. The main peaks of Bi 4f_7/2_ and Bi 4f_5/2_ of DP are located at binding energies of 159.00 and 164.33 eV, respectively. Beyond the two main peaks, assigned to the Bi^3+^ ions in the perovskite, all of the composites exhibit two extra spin–orbit doublets which are assigned to elemental bismuth Bi(0) from bismuthene. The XPS survey spectrum of bismuthene (Figure S3) indicates the presence of Bi, O, and C. The Bi 4f spectrum of bismuthene (Figure 6a) was deconvoluted into two spin–orbit doublets centered at 156.49 and 161.80 eV, which are typically attributed to Bi–Bi bonds of elemental Bi(0) 4f_7/2_ and Bi(0) 4f_5/2_,^38^ respectively, while the peaks at 158.64 and 163.95 eV were assigned to Bi–O 4f_7/2_ and Bi–O 4f_5/2_ originating from the partially oxidized bismuthene surface.^24,39^ It has been reported that the partial surface oxidation of bismuthene works as a passivation layer that actually prevents further inner degradation, in the same way that aluminum oxide prevents the oxidation of deeper aluminum.^40^Figure 6a shows that the peaks of Bi(0) 4f_5/2_ and Bi(0) 4f_7/2_ for the composites are shifted in comparison to those for the bismuthene sample, reaching a shift by ca. 1.46 and 1.26 eV for Bi(0) 4f_5/2_ and Bi(0) 4f_7/2_ in DP/Bi2, respectively, indicating interfacial interactions between the Bi atoms in bismuthene and the DP nanoparticles.^34^

Figure 6b shows the O 1s spectra of DP and DP/Bi composites. The O 1s spectrum of DP is deconvoluted into two peaks centered at 532.32 and 533.84 eV attributed to the residual organic C–O and C=O bonds, respectively, from the capping oleic acid used in the synthesis process. Beyond the peaks of C–O and C=O bonds, the O 1s spectra of all the composites exhibit a third peak at binding energies of 530.17, 530.17, and 530.24 eV for DP/Bi0.5, DP/Bi1, and DP/Bi2, respectively, which is assigned to the Bi–O bond, which is further evidence of the bismuthene presence in the composites.

Table 1 shows that almost all core levels in the composites exhibited a positive shift in the binding energy compared with those transitions in the pristine perovskite that slightly increase with the amount of bismuthene in the composite. For DP/Bi2, the Bi^3+^ 4f_7/2_ and Bi^3+^ 4f_5/2_ peaks (Figure 6a) both shifted by ca. 0.14 eV; the Cs 3d_5/2_ and Cs 3d_3/2_ peaks (Figure 6c) shifted by 0.16 and 0.17 eV, respectively; Ag 3d_5/2_ and Ag 3d_3/2_ (Figure 6d) shifted by 0.13 and 0.12 eV, respectively. Unlike the other atoms, Br 3d_5/2_ and Br 3d_3/2_ (Figure 6e) exhibited a lower shift by ca. 0.09 and 0.08 eV, respectively, compared with the pristine perovskite. The lower and higher shifts of the binding energies of Cs 3d, Ag 3d, and Bi 4f in DP/Bi composites indicate that there has been a change in the electronic density of Cs_2_AgBiBr_6_ and bismuthene in DP/Bi composites, which may be attributed to strong Bi–Cs, Bi–Ag, and Bi–Bi interfacial interactions.^34^ The C 1s spectra of DP and the DP/Bi composites (Figure S4) were deconvoluted into three peaks. The main peak at 284.8 eV was assigned to the aliphatic C–C bond from the residual oleic acid used in the synthesis of the samples and was used to calibrate the XPS spectra. The 286.38 and 289.38 eV peaks in DP confirmed the presence of the C–O and O=C–O bonds, respectively, from oleic acid. The position of the peaks for the C–O and O=C–O bonds in the composites can be observed in Table 1. The main peak of the C 1s spectra of Bi (Figure S5) was assigned to the C–C bond from adventitious carbon and was used as a reference for binding energy values.

**Figure 7:**
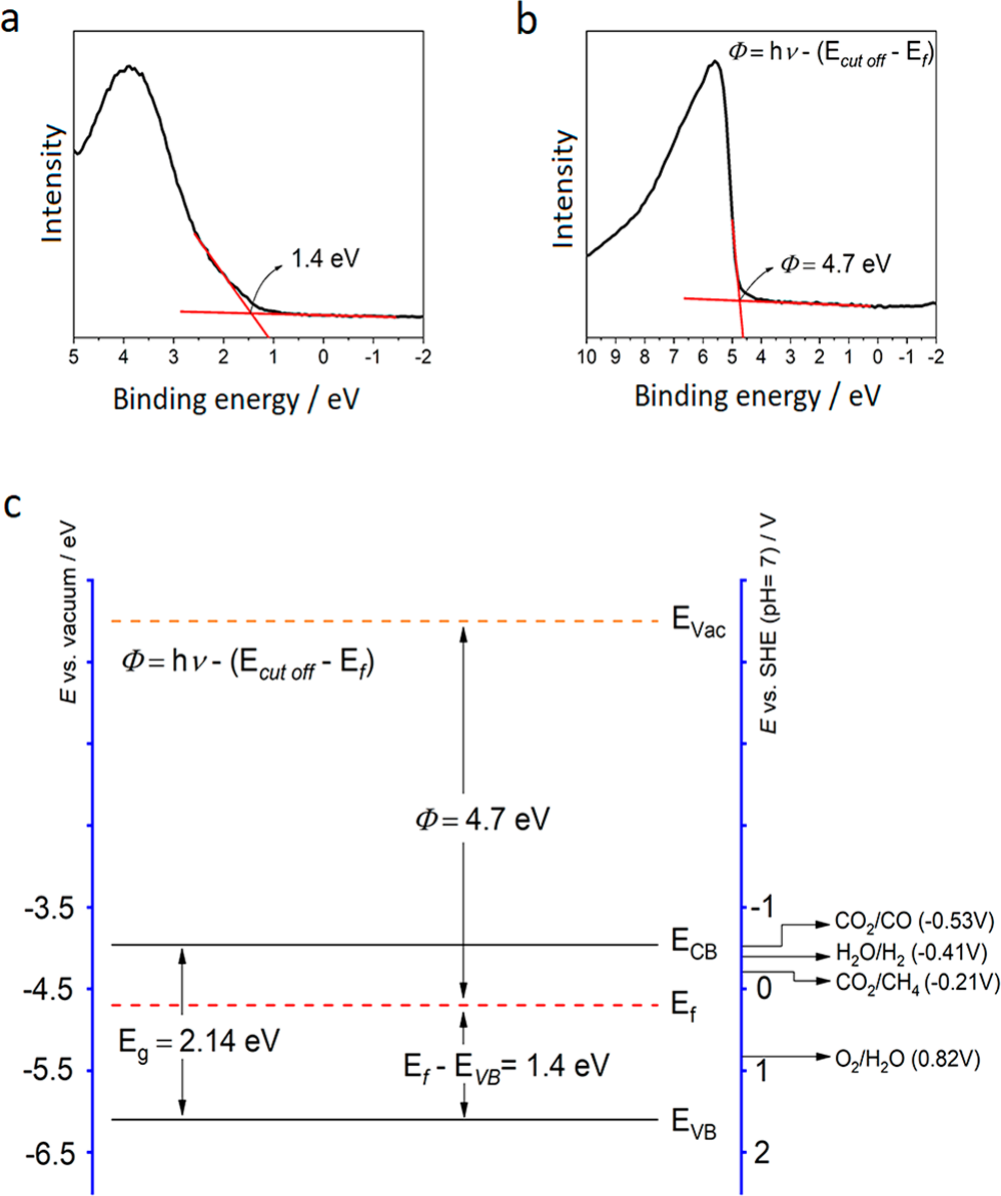
(a) Valence band XPS, (b) electronic work function, and (c) energy band diagram of Cs_2_AgBiBr_6_ where *E*_g_, *E*_f_, Φ, *E*_VB_, and *E*_CB_ represent the band gap energy, Fermi level, electronic work function, valence band edge, and conduction band edge, respectively.

The valence band (VB) XPS was used to determine the energy gap between the VB edge and the Fermi level (*E*_f_) (Figure 7a). The value measured from the intercept between the tangent of the onset and the baseline of the spectra was approximately 1.40 eV, which is consistent with the VB energy value of the mechanochemically synthesized Cs_2_AgBiBr_6_ reported by Kumar et al.^22^

Additionally, the electronic work function, i.e., the energy difference between the *E*_f_ and the vacuum level, was measured to be 4.70 eV (Figure 7b). Combining this information with the band gap (2.14 eV) from the UV–vis DRS (see Figure 3a), which is defined as the energy gap between the CB and VB, the electronic band diagram of Cs_2_AgBiBr_6_ was constructed, as can be seen in Figure 7c.

The as-prepared Cs_2_AgBiBr_6_ nanoparticles exhibited a CB edge potential of −3.96 eV from the vacuum level (−0.54 V vs SHE at pH 7). These results suggest that the DP nanoparticles have a suitable CB edge potential with a sufficiently shallow potential to allow them to overcome the electrochemical potential to form multielectron reduced species from CO_2_, such as CH_4_ (CO_2_/CH_4_, −0.21 V vs SHE at pH 7) and CO (CO_2_/CO, −0.53 V vs SHE at pH 7).^41^

**Figure 8:**
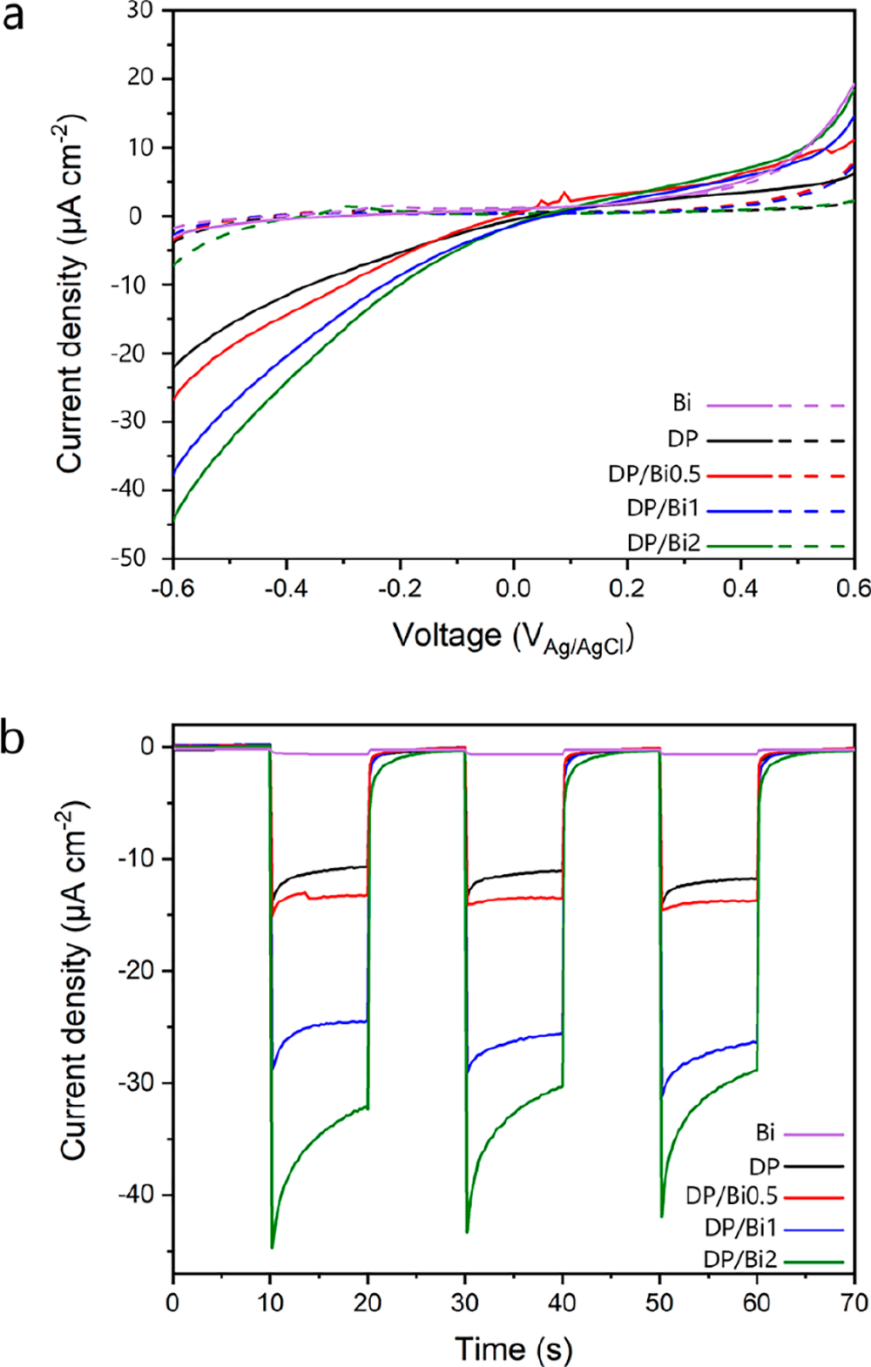
(a) Linear sweep voltammetry measurements of Bi, DP, DP/Bi0.5, DP/Bi1, and DP/Bi2 photoelectrodes in darkness (dashed) and under light (solid) illumination of 100 mW cm^–2^ in 0.1 M TBAPF_6_ in acetonitrile solution. (b) Transient photocurrent response of Bi, DP, DP/Bi0.5, DP/Bi1, and DP/Bi2 photoelectrodes under on–off cycles of light illumination (100 mW cm^–2^) at −0.4 V vs Ag/AgCl in 0.1 M TBAPF_6_ in acetonitrile solution.

Since the mobility and efficient separation of the photogenerated charges play a pivotal role in the CO_2_ reduction reaction, the photoelectrochemical (PEC) behavior of the samples was evaluated by measuring their photocurrent response. Figure 8a displays the current–voltage characteristics of Bi, DP, DP/Bi0.5, DP/Bi1, and DP/Bi2 in the dark and under illumination.

The linear sweeps from −0.6 to +0.6 V vs Ag/AgCl in the dark show a very small current density in the range of 10^–6^ A cm^–2^ and an increasing photocurrent response with growing bismuthene content at a negative applied bias under illumination. Figure 8b exhibits the transient photocurrent measurements carried out under chopped light illumination with a fixed bias of −0.4 V vs Ag/AgCl, showing immediate photocurrent generation in the DP photoelectrode under light irradiation, reaching a current density of 10.6 μA cm^–2^.

The current density increased with the increase of the bismuthene amount in the composites, measuring 13.2, 24.3, and 32.3 μA cm^–2^ at −0.4 V (vs Ag/AgCl) for DP/Bi0.5, DP/Bi1, and DP/Bi2, respectively. These results show that the PEC performance is significantly improved by the presence of bismuthene, with DP/Bi2 reaching 3 times higher photocurrent density compared to bare DP, suggesting that bismuthene improved the separation of photoexcited electrons and holes in the perovskite.^42^ These results are in agreement with the interfacial interactions observed by XPS in Figure 6. This agrees with our previously reported results on BiVO_4_/bismuthene/NiFeOOH composite photoanodes for PEC water oxidation, where composites with bismuthene showed a much higher current density compared to the bare BiVO_4_ photoanode due to improved band bending.^25^ Bismuthene, therefore, increases the separation and extraction of photogenerated charges.

**Figure 9:**
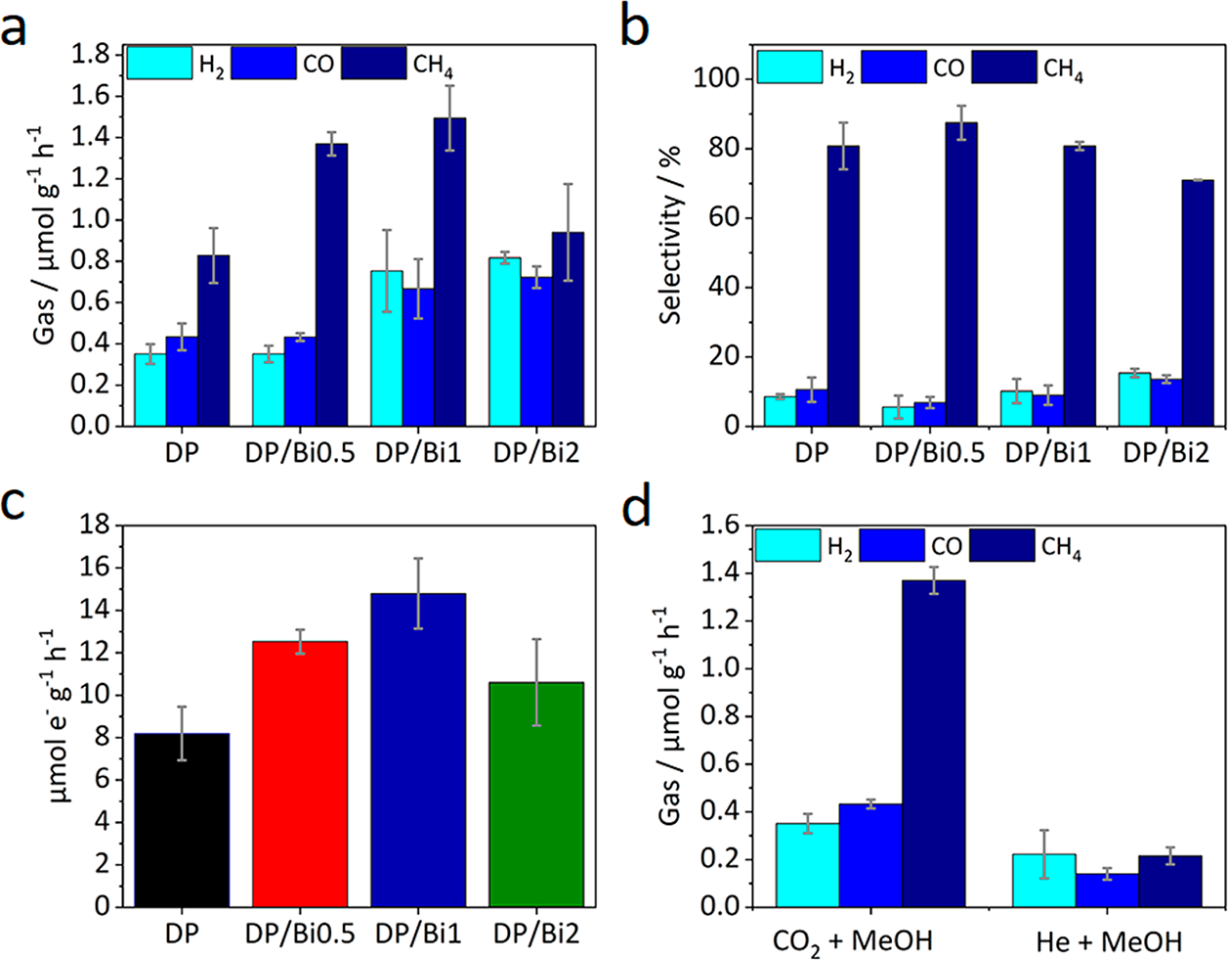
(a) H_2_, CO, and CH_4_ production rates from photocatalytic CO_2_ reduction in the liquid phase using methanol suspensions of different photocatalysts under simulated solar light for 2 h. (b) Calculated selectivity of the obtained products. (c) Total electron e^–^ consumption from the photocatalytic CO_2_ reduction. (d) Photocatalytic results under different reaction conditions for a representative DP/Bi0.5 sample.

Finally, the photocatalytic performance of the samples in the CO_2_ reduction reaction was evaluated in the liquid phase using methanol as a hole scavenger and proton source. The reaction was carried out under simulated solar light, 1 sun (100 mW cm ^–2^) with no cutoff filter, and the results are presented in Figure 9. The photocatalytic activity of the samples is presented in terms of production rates of H_2_, CO, and CH_4_ gases in μmol g^–1^ h^–1^ (Figure 9a). The photocatalytic production rates of Cs_2_AgBiBr_6_ (DP) nanoparticles are 0.83(±0.13) μmol g^–1^ h^–1^ CH_4_, 0.43(±0.06) μmol g^–1^ h^–1^ CO, and 0.35(±0.05) μmol g^–1^ h^–1^ H_2_.

The DP/Bi0.5 composite increased the CH_4_ production by 65% in comparison with pure DP, while CO and H_2_ productions remained constant with production rates of 1.37(±0.06) μmol g^–1^ h^–1^ CH_4_, 0.43(±0.02) μmol g^–1^ h^–1^ CO and 0.35(±0.04) μmol g^–1^ h^–1^ H_2_. When the bismuthene content was increased in DP/Bi1, the CH_4_ production had a slight increase of 8% compared with DP/Bi0.5, while CO and H_2_ production rates increased by 55% and 114%, respectively, reaching the values of 1.49(±0.16) μmol g^–1^ h^–1^ CH_4_, 0.67(±0.14) μmol g^–1^ h^–1^ CO, and 0.75(±0.20) μmol g^–1^ h^–1^ H_2_. When the bismuthene content was further increased in DP/Bi2, the CH_4_ production rate decreased by 63% compared with DP/Bi1, but CO and H_2_ productions slightly increased by 7% and 9%, respectively, showing a production of 0.94(±0.23) μmol g^–1^ h^–1^ CH_4_, 0.72(±0.05) μmol g^–1^ h^–1^ CO, and 0.82 ±0.03) μmol g^–1^ h^–1^ H_2_.

The selectivity of CO, CH_4_ and H_2_ was calculated using eqs 1–3, respectively, and the results are presented in Figure 9b. DP/Bi0.5 showed the highest selectivity for CH_4_, 87(±5)%, compared with 81(±7)% CH_4_ selectivity for pristine DP. The composite DP/Bi1 showed the same selectivity for CH_4_ as DP of 81(±1)%, and as the bismuthene content increased in DP/Bi2, the CH_4_ selectivity decreased to 71(±1)%.

Figure 9c shows the photocatalytic activity of the samples on an electron basis considering the eight-electron formation of CH_4_, two-electron formation of CO, and two-electron formation of H_2_. Cs_2_AgBiBr_6_ (DP) showed a photocatalytic activity of 8.19 μmol e^–^ g^–1^ h^–1^, DP/Bi0.5 of 12.53 μmol e^–^ g^–1^ h^–1^, DP/Bi1 of 14.79 μmol e^–^ g^–1^ h^–1^, and DP/Bi2 of 10.61 μmol e^–^ g^–1^ h^–1^. These results show that DP/Bi1 had higher overall photocatalytic activity compared to the other samples and confirm that the photocatalytic activity increases with increasing bismuthene content. This is consistent with the higher current density and improved separation of the photogenerated charges in the composites compared with the pristine perovskite (see Figure 8b), reaching the optimal amount of 1% of bismuthene and decreasing when the bismuthene amount is further increased to 2%.

These results agree with what is observed in the literature on the electroreduction reaction of CO_2_ by bismuthene, where it is reported that the stacking of layered bismuthene, forming a compact layer catalyst, decreases the availability of active sites for reactants in the bismuthene nanosheets. Thus, the thick bismuthene nanosheets have lower activity and stability relative to thin bismuthene.^24^ In this work, the higher content of bismuthene in DP/Bi2 can generate stacking of bismuthene nanosheets, which can explain the reduction of the photocatalytic activity and the selectivity for CH_4_.

The photocatalytic activity in terms of total electron consumption and the CH_4_ selectivity of DP and DP/Bi samples was also compared with other reported halide perovskite-based photocatalysts and is summarized in Table S1. The Cs_2_AgBiBr_6_ and Cs_2_AgBiBr_6_/bismuthene composites in this work achieved higher photocatalytic activity than previously reported Cs_3_Sb_2_I_9_ photocatalysts^43^ and showed higher selectivity to CH_4_ than most of the other halide perovskite based composites analyzed in Table S1.

The photocatalytic CO_2_ reduction was confirmed by a series of control experiments such as experiments (1) in darkness, (2) without photocatalyst, and (3) in He. No detectable products were found in the experiments carried out in the dark or without photocatalyst, indicating that the CO_2_ reduction reaction takes place as a light-driven catalytic process involving the photocatalysts.^44^Figure 9d shows the photocatalytic results in the reactions carried out with and without CO_2_. In the reaction with He replacing CO_2_, small amounts of H_2_ (0.22 ± 0.10 μmol g^–1^ h^–1^) were detected, which can be attributed to the photocatalytic dehydrogenation of methanol.^45,46^ Trace amounts of CO and CH_4_, 0.14 ± 0.02 and 0.22 ± 0.04 μmol g^–1^ h^–1^, respectively, were also detected, presumably from the slight decomposition of the organic ligands capping the photocatalysts and the presence of preadsorbed CO_2_ on the photocatalyst. The drastic drop in production rates of carbon products in the reaction without CO_2_ indicates that the overall reaction involves CO_2_ reduction and methanol oxidation cycles to produce CH_4_, and CO, whereas H_2_ is formed in a parallel competitive methanol reduction reaction.

To assess the stability of the composite in the photocatalytic CO_2_ reduction, two sequential runs of 2 h each were performed on a representative DP/Bi1 sample (Figure S6). After one cycle, the production rates change from 1.24 to 0.03 μmol g^–1^ h^–1^ CH_4_, from 0.55 to 0.03 μmol g^–1^ h^–1^ CO, and from 0.61 to 0.05 μmol g^–1^ h^–1^ H_2_. The XRD patterns and SEM micrographs of DP/Bi1 before and after two cycling tests (Figures S7 and S8) show that there is some crystal structure and morphological change during the photocatalytic reaction. The XRD patterns for the sample after the photocatalytic reaction show narrower cubic Cs_2_AgBiBr_6_ peaks along with minor secondary peaks, which correspond to the ternary phase Cs_3_Bi_2_Br_9_ (COD 96-210-6377). These changes indicate that, during the photocatalytic reaction, part of Cs_2_AgBiBr_6_ sintered into larger diffraction domains and another part decomposed into Cs_3_Bi_2_Br_9_, resulting in a drop in photocatalytic activity after the first run test. Therefore, these composites would benefit from strategies to improve their stability, such as using reduced graphene oxide,^22^ nitrogen-doped carbon^47^ or Ce-UiO-66-H^48^ as a protective layer, which would give it a more hydrophobic character, which will be addressed in future work.

The apparent quantum efficiency (AQE) of CH_4_ production of the DP/Bi1 sample was calculated to describe the efficiency of the photocatalysts in absorbing incident photons and generating photoinduced charges that react to produce solar fuels. The calculated AQE for DP/Bi1 was 0.0012% at 450 nm.

From these observations, it can be inferred that CH_4_, CO, and H_2_ were produced via the following reaction mechanism: Cs_2_AgBiBr_6_ is photoexcited, generating holes (h^*+*^) that migrate to its surface and electrons (e^*–*^) that migrate and are collected on the bismuthene nanosheets
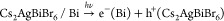
5

The photogenerated holes, h^*+*^, oxidize methanol to produce hydroxymethyl radical, ^•^CH_2_OH, and H^+^ ^49^
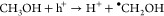
6

At this point, parallel reduction reactions can take place, such as the two-electron reduction of CO_2_ to produce CO^50^
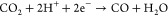
8the eight-electron reduction of CO_2_ to produce CH_4_^50^
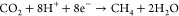
9or the competitive two-electron reduction of the protons H^+^, originating from the methanol oxidation, producing H_2_^41^
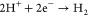
10

The results indicate that the low concentration of bismuthene in the composites, as in DP/Bi0.5, favored reaction 9, thereby increasing the production of CH_4_ and not altering CO and H_2_ production. As the bismuthene content was increased in DP/Bi1, reactions 8 and 10 were favored, thereby increasing the production of CO and H_2_, with the CH_4_ production remaining almost unchanged. When the bismuthene content was further increased in DP/Bi2, CH_4_ production decreased, and CO and H_2_ production remained almost unchanged. This can be explained by the bismuthene having assisted the separation of photogenerated electron–hole pairs from the perovskite, thereby providing a higher availability of electrons for the multielectron reduction of CO_2_ to CH_4_. However, the higher concentration of bismuthene in the composites increased the selectivity toward CO and H_2_ production.

## Conclusions

We successfully synthesized Cs_2_AgBiBr_6_ double perovskite (DP) nanoparticles and their composites with bismuthene (Bi) by a simple and fast ligand-assisted reprecipitation method. The resulting DP/Bi composites exhibited significantly improved photocatalytic activity for CO_2_ reduction using methanol as a hole scavenger, showing a CH_4_ production of up to 1.49(±0.16) μmol g^–1^ h^–1^ (under 1 sun) with a CH_4_ selectivity of 81(±1)% for an optimized DP/Bi composite with 1 wt % bismuthene. This result was 80% higher than that for Cs_2_AgBiBr_6_ nanoparticles. The higher performance of the DP/Bi composite for photocatalytic CO_2_ reduction shows that the synthesis method adopted in this work involving a ligand-assisted reprecipitation method proved to be efficient in creating a good interfacial contact between the Cs_2_AgBiBr_6_ and bismuthene, which promotes efficient photoinduced charge carrier separation and, consequently, a lower recombination of electron–hole pairs. This was confirmed by photoelectrochemical measurements. The improved optical and electronic properties of the composites allowed a higher absorption of the light energy and facilitated migration of the electrons to the bismuthene layers and holes to the Cs_2_AgBiBr_6_ surface, thereby improving its overall photocatalytic activity. This strategy shows great potential for creating two-dimensional Pb-free halide perovskites on a larger scale with improved CH_4_ selectivity for solar fuels production.

## Data Availability

The data that support the findings of this study are openly available in the Imperial College London research data repository at https://doi.org/10.14469/hpc/12213.

## Supporting Information Available

XPS spectra, summary of photocatalytic CO_2_ reduction performances, photocatalytic activity results, powder XRD patterns, SEM micrographs, and apparent quantum efficiency calculation (PDF)

## Abbreviations

LARP: ligand-assisted reprecipitation
PEC: photoelectrochemical
TBAPF_6_: tetrabutylammonium hexafluorophosphate
DMF: dimethylformamide
DI: deionized
AQE: apparent quantum efficiency
CB: conduction band
VB: valence band
MeOH: methanol
