# Supplementary material for: Lead-Free Halide Perovskite Cs2AgBiBr6/Bismuthene Composites for Improved CH4 Production in Photocatalytic CO2 Reduction
Source: ACS Appl Energy Mater. 2023 Feb 1;6(20):10193–204. doi: 10.1021/acsaem.2c03105 (PMC10598630; doi:10.1021/acsaem.2c03105)
Supplement: Supplementary file 1 — ae2c03105_si_001.pdf [file ae2c03105_si_001.pdf]

Supporting Information

Lead-Free Halide Perovskite $Cs_2AgBiBr_6$/Bismuthene Composites for Improved CH_4 Production in Photocatalytic CO_2 Reduction

Michael Segundo Sena,[†, ‡] Junyi Cui,[‡] Yasmine Baghdadi,[‡] Eduardo Rattner,[‡] Matyas Daboczi,[‡] André Luís Lopes-Moriyama,[†] Andarair Gomes dos Santos,[§] and Salvador Eslava[*, ‡]

[†] Department of Graduation in Chemical Engineering, Universidade Federal do Rio Grande do Norte/UFRN, 59.078-970 Rio Grande do Norte, Brazil. E-mail: michael.sena.020@ufrn.edu.br

[‡] Department of Chemical Engineering, Imperial College London, SW7 2BX, London, United Kingdom. E-mail: s.eslava@imperial.ac.uk

[§] Department of Agrotechnology and Social Sciences, Universidade Federal Rural do Semi-Árido/UFERSA, 59.600-000 Rio Grande do Norte, Brazil.

*Corresponding author. E-mail address: s.eslava@imperial.ac.uk

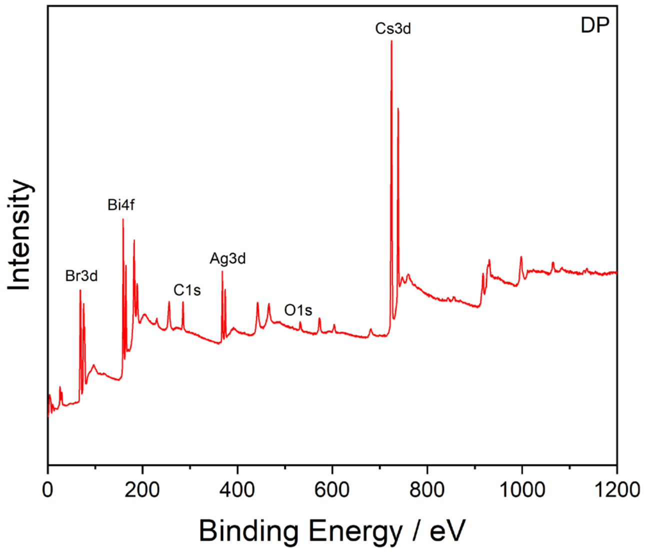

Figure S1. XPS survey spectrum of $Cs_2AgBiBr_6$ (DP).

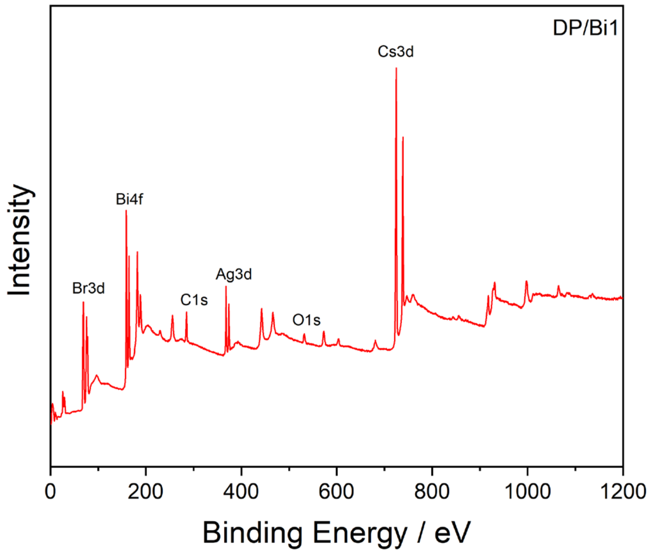

Figure S2. XPS survey spectrum of $Cs_2AgBiBr_6$/bismuthene composite with nominal 1 wt% of bismuthene (DP/Bi1).

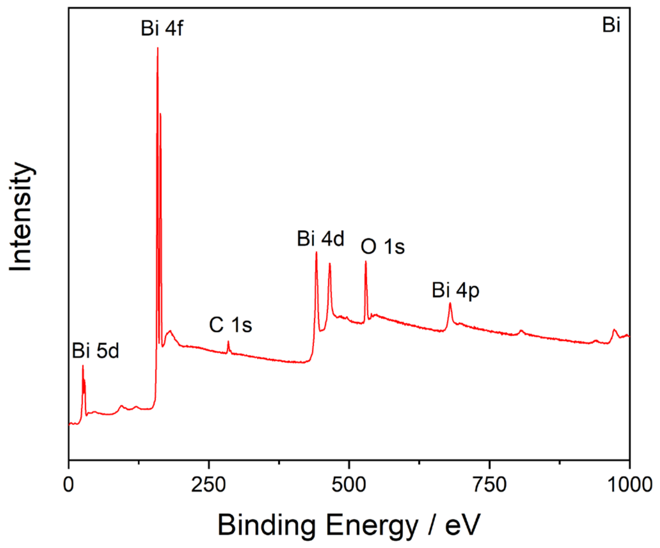

Figure S3. XPS survey spectrum of bismuthene (Bi).

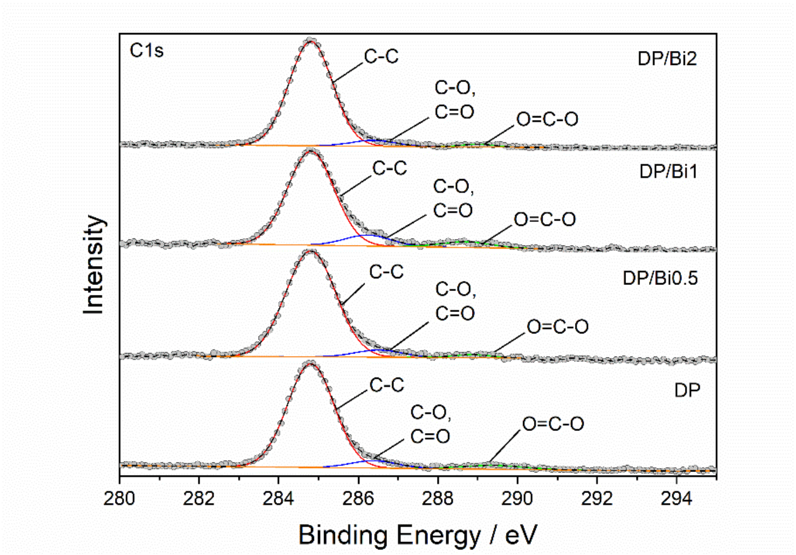

Figure S4. C *1s* spectra of $Cs_2AgBiBr_6$ (DP) and $Cs_2AgBiBr_6$–bismuthene (DP/Bi) composites.

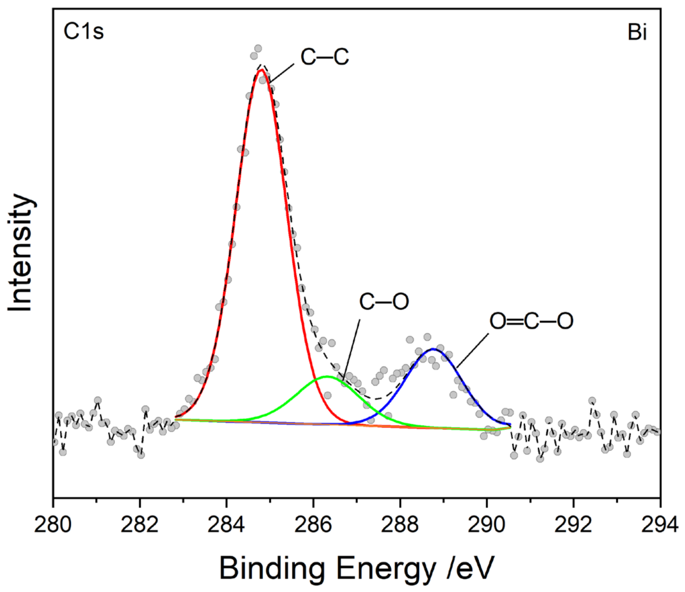

Figure S5. C *1s* spectrum of bismuthene (Bi).

Table S1. Summary of the photocatalytic CO_2 reduction performances of $Cs_2AgBiBr_6$ (DP), $Cs_2AgBiBr_6$/bismuthene (DP/Bi) composites and other reported halide perovskite-based photocatalysts.

Photocatalyst	Total electron consumption (μmol g^{-1} h^{-1})	CH_4 selectivity (%)	Medium	Light source	Reference
DP<br>DP/Bi0.5<br>DP/Bi1<br>DP/Bi2	8.19<br>12.53<br>14.79<br>10.61	81<br>87<br>81<br>71	methanol	300 W Xe (AM 1.5G), 100 mW cm^{-2}	This work
$Cs_3Sb_2I_9$	4.7	0 (100 % for CO)	CO_2 (g) water vapor	300 W Xe (λ ≥ 420 nm)	1
$CsPbBr_3$/Cu/RGO	103	98.5	CO_2 (g) water vapor	300 W Xe (AM 1.5G), 100 mW cm^{-2}	2
$Cs_2AgBiBr_6$/CTF	130.2	53	ethyl acetate	300 W Xe (λ ≥ 420 nm)	3
$CsPbBr_3$/AgBr	141.4	43	acetonitrile/ water	300 W Xe (λ ≥ 420 nm)	4
$Cs_2AgBiBr_6$/Ce-UiO-66-H	311.8	1 (99 % for CO)	CO_2 (g) water vapor	300 W Xe	5
$Cs_3Bi_2I_9/CeO_2$	73	-	CO_2 (g) water vapor	300 W Xe	6
MCM-41@$Cs_3Bi_2Br_9$	34.5	0 (100 % for CO)	CO_2 (g) water vapor	300 W Xe (λ ≥ 420 nm), 350 mW cm^{-2}	7
$CsPbBr_3$/BIF-122-Co	52.2	81	ethyl acetate/water	300 W Xe (λ ≥ 420 nm)	8
$Ti_3C_2/CsPbBr_3$ QD	190.4	81.1	ethyl acetate	300 W Xe	9

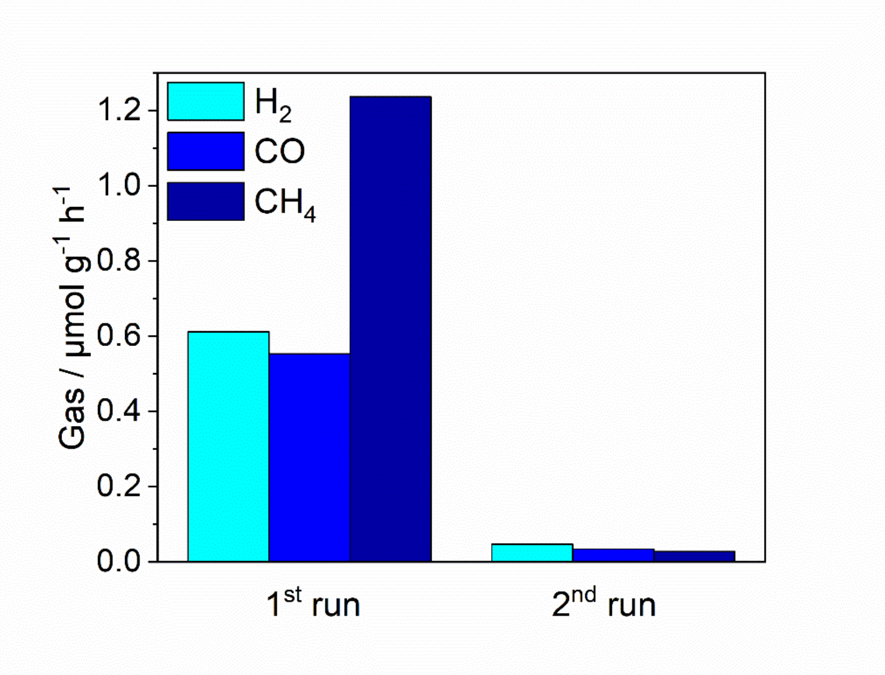

Figure S6. Recycling test for the photocatalytic activity of a representative DP/Bi1.

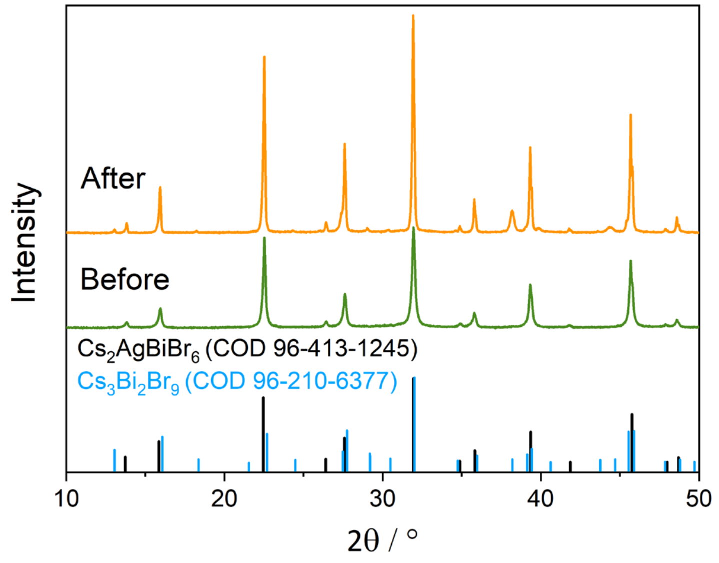

Figure S7. XRD patterns of DP/Bi1 before and after CO_2 photocatalytic conversion.

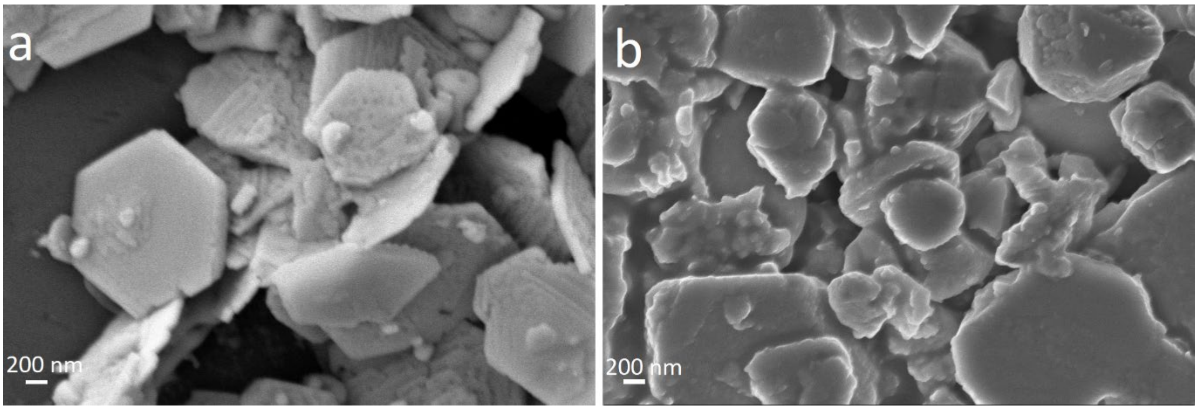

Figure S8. SEM micrographs of DP/Bi1: (a) before and (b) after CO_2 photocatalytic conversion.

Apparent Quantum Efficiency Calculations

The apparent quantum yield efficiency (AQE) was measured using the same experimental setup but using a 450 nm LED light source to obtain a monochromatic light. The AQE equation is represented below:[10]

$$AQE/\% = \frac{\text{Number of reacted electrons}}{\text{Number of incident photons}} \text{ x } 100$$

Number of incident photons is calculated using the following equations:

$$\text{Number of moles of incident photons per time } (N_{Einstein}) = \frac{\text{Number of incident photons per time } (\text{N}_\text{P})}{\text{N}_\text{A}}$$

Where N_P can be calculates as follows:

$$\text{N}_\text{P} = \frac{\text{Light Intensity (E)}}{\text{Photon Energy } (\text{E}_\text{P})} \text{ and Photon Energy } (\text{E}_\text{P}) = \frac{\text{hc}}{\lambda}$$

Substituting, we obtain:

$$\text{E}_\text{P} = \frac{(6.625 \text{ x } 10^{-34} \text{ J s}) \, (3 \text{ x } 10^{17} \text{ nm s}^{-1})}{450 \text{ nm}} = 4.42 \text{ x } 10^{-19} \text{ J} ;$$

Light intensity (E) = Irradiance (W m^{-2}) x effective light irradiation area (m^2).

The irradiance inside the reactor was measured using a light intensity meter. The measured irradiance in the reactor was 100 mW cm^{-2} and the effective light irradiation area was 19.63 cm^2, therefore the calculated *E* was 1.96 W or J s^{-1} at 450 nm.

Substituting, we obtain:

$$\text{N}_\text{P} = \frac{\text{E}}{\text{E}_\text{P}} = \frac{1.96 \text{ J s}^{-1}}{4.19 \text{ x } 10^{-19} \text{J}} = 4.45 \text{ x } 10^{18} \text{s}^{-1}$$

$$\text{N}_\text{Einstein} = \frac{\text{N}_\text{P}}{\text{N}_\text{A}} = \frac{4.45 \text{ x } 10^{18} \text{s}^{-1}}{6.022 \text{ x } 10^{23} \text{mol}^{-1}} = 7.38 \text{ x } 10^{-6} \text{ mol s}^{-1} = 7.38 \text{ µmol s}^{-1}$$

The CH_4 production was 0.04 µmol h^{-1} with 450 nm monochromatic light under experimental conditions. Finally,

$$AQE \text{ } (\%) = \frac{8 \text{ x } \text{CH}_4 \text{ production rate in µmol s}^{-1}}{\text{N}_\text{Einstein} \text{in µmol s}^{-1}} \text{ x } 100 = \frac{8 \text{ x } 1.11 \text{ x } 10^{-5} \text{µmol s}^{-1}}{7.38 \text{ µmol s}^{-1}} \text{ x } 100 = 0.0012 \text{ } \%$$

References

(1) Hai, Y.; Huang, W.; Li, Z.; Wu, D.; Huang, Q.; Tang, X. Morphology Regulation and Photocatalytic CO_2 Reduction of Lead-Free Perovskite $Cs_3Sb_2I_9$ Microcrystals. *ACS Appl. Energy Mater.* **2021**, *4* (6), 5913–5917. https://doi.org/10.1021/acsaem.1c00722.

(2) Kumar, S.; Regue, M.; Isaacs, M. A.; Freeman, E.; Eslava, S. All-Inorganic $CsPbBr_3$ Nanocrystals: Gram-Scale Mechanochemical Synthesis and Selective Photocatalytic CO_2 Reduction to Methane. *ACS Appl. Energy Mater.* **2020**, *3* (5), 4509–4522. https://doi.org/10.1021/acsaem.0c00195.

(3) Zhang, Z.; Jiang, Y.; Dong, Z.; Chu, Y.; Xu, J. 2D/2D Inorganic/Organic Hybrid of Lead-Free $Cs_2AgBiBr_6$ Double Perovskite/Covalent Triazine Frameworks with Boosted Charge Separation and Efficient CO_2 Photoreduction. *Inorg. Chem.* **2022**, *61* (40), 16028–16037. https://doi.org/10.1021/acs.inorgchem.2c02440.

(4) Zhang, Z.; Dong, Z.; Jiang, Y.; Chu, Y.; Xu, J. A Novel S-Scheme Heterojunction of $CsPbBr_3$ Nanocrystals/AgBr Nanorods for Artificial Photosynthesis. *Chem. Eng. J.* **2022**, *435* (P2), 135014. https://doi.org/10.1016/j.cej.2022.135014.

(5) Ding, L.; Bai, F.; Borjigin, B.; Li, Y.; Li, H.; Wang, X. Embedding $Cs_2AgBiBr_6$ QDs into Ce-UiO-66-H to in Situ Construct a Novel Bifunctional Material for Capturing and Photocatalytic Reduction of CO_2. *Chem. Eng. J.* **2022**, *446* (P2), 137102. https://doi.org/10.1016/j.cej.2022.137102.

(6) Feng, Y. X.; Dong, G. X.; Su, K.; Liu, Z. L.; Zhang, W.; Zhang, M.; Lu, T. B. Self-Template-Oriented Synthesis of Lead-Free Perovskite $Cs_3Bi_2I_9$ Nanosheets for Boosting Photocatalysis of CO_2 Reduction over Z-Scheme Heterojunction $Cs_3Bi_2I_9/CeO_2$. *J. Energy Chem.* **2022**, *69*, 348–355. https://doi.org/10.1016/j.jechem.2022.01.015.

(7) Cui, Z.; Wang, P.; Wu, Y.; Liu, X.; Chen, G.; Gao, P.; Zhang, Q.; Wang, Z.; Zheng, Z.; Cheng, H.; Liu, Y.; Dai, Y.; Huang, B. Space-Confined Growth of Lead-Free Halide Perovskite $Cs_3Bi_2Br_9$ in MCM-41 Molecular Sieve as an Efficient Photocatalyst for CO_2 Reduction at the Gas−solid Condition under Visible Light. *Appl. Catal. B Environ.* **2022**, *310* (April), 121375. https://doi.org/10.1016/j.apcatb.2022.121375.

(8) Chen, Z.; Hong, Q.; Zhang, H.-X.; Zhang, J. Composite of $CsPbBr_3$ with Boron Imidazolate Frameworks as an Efficient Visible-Light Photocatalyst for CO_2 Reduction. *ACS Appl. Energy Mater.* **2022**, *5* (1), 1175–1182. https://doi.org/10.1021/acsaem.1c03552.

(9) Zhang, Y.; Chen, W.; Zhou, M.; Miao, G.; Liu, Y. Efficient Photocatalytic CO_2 Reduction by the Construction of $Ti_3C_2/CsPbBr_3$ QD Composites. *ACS Appl. Energy Mater.* **2021**, *4* (9), 9154–9165. https://doi.org/10.1021/acsaem.1c01406.

(10) Kumar, S.; Regue, M.; Isaacs, M. A.; Freeman, E.; Eslava, S. All-Inorganic $CsPbBr_3$ Nanocrystals: Gram-Scale Mechanochemical Synthesis and Selective Photocatalytic CO_2 Reduction to Methane. *ACS Appl. Energy Mater.* **2020**, *3* (5), 4509–4522. https://doi.org/10.1021/acsaem.0c00195.
